# Viral encephalitis caused by respiratory viruses

**DOI:** 10.1186/1471-2334-13-S1-P78

**Published:** 2013-12-16

**Authors:** Angelica Vişan, Monica Luminos, Magda Vasile, Anca Drăgănescu, Gheorghiță Jugulete, Anuța Bilaşco, Cristina Negulescu, Camelia Kouris, Ruxandra Măntescu, Mădălina Maria Merişescu, Endis Osman, Sabina Șchiopu, Dragoş Florea, Dan Oțelea

**Affiliations:** 1 National Institute for Infectious Diseases “Prof. Dr. Matei Balş”, Bucharest, Romania; 2 Carol Davila University of Medicine and Pharmacy Bucharest, Romania

## Background

Viral encephalitis is a relatively rare disease but with a potentially high morbidity and mortality. Advances in the diagnostic methods can lead to the identification of a large number of viruses that can affect the central nervous system.

## Methods

We conducted a retrospective, comparative survey of the pediatric cases of viral encephalitis caused by respiratory viruses that were submitted at the National Institute for Infectious Diseases “Prof. Dr. Matei Balş” over the last 2 years (2011-2013).

## Results

Using molecular diagnostic methods (PLEX-ID, Multiplex PCR) we identified, in the respiratory secretions and in the CSF: H1N1 influenza virus (2009), influenza AH3 virus, influenza B virus, coronavirus and adenovirus.

The most severe forms of the disease were caused by the influenza virus H1N1. There were 2 cases of encephalitis admitted with this etiology, one of which had a fatal outcome and the other progressed to neurological decline. Other cases of encephalitis, having as etiology other respiratory viruses, have evolved favorably, with full recovery without sequelae.

## Conclusion

We emphasize the importance of using molecular diagnostic tests which can provide rapid diagnosis and lead to appropriate therapeutic strategies and epidemiological measures.

